# Assessment of blood pressure measurement skills in second-year medical students after ongoing simulation-based education and practice

**DOI:** 10.1080/10872981.2020.1841982

**Published:** 2020-11-01

**Authors:** Yuka Yamazaki, Iku Hiyamizu, Kyoko Joyner, Junji Otaki, Yukie Abe

**Affiliations:** a Department of Medical Education, Tokyo Medical University, Shinjuku-ku, Tokyo, Japan; b Simulation Center, Tokyo Medical University Hospital, Shinjuku-ku, Tokyo, Japan

**Keywords:** Blood pressure, assessment, simulation, medical students, clinical skills

## Abstract

**Background:**

Simulation-Based Education (SBE) simulates specific physiological characteristics of a patient, allowing student practice in developing clinical skills and assessment of skill competency. Literature is limited regarding SBE’s effectiveness in curriculum enrichment. This study investigated Blood Pressure (BP) measuring proficiency of second-year medical students with first-year SBE training and a second-year review, by comparing data from Simulation-Based assessments in 2017 and 2019.

**Methods:**

Second-year medical students measured BP on three manikin arms, associated with distinct clinical contexts (healthy young male, young female experiencing hypotension, and older male suffering hypertension and diabetes). All manikins’ BP settings were independent of clinical context. In January 2019, 108 second-year medical students who received traditional training, as well as SBE in 2017 and Simulation-Based practice in 2018, were divided into four groups (n = 32, 24, 24, and 28), with two groups each assessed on consecutive days. The proportions of correct BP values in each of three contexts were compared between experiments in 2017 and 2019. Additionally, systolic (SBP) and diastolic (DBP) blood pressure results were stratified into three groups: lower than setting value, correct, and higher than setting vgalue, with proportions for each group compared for the 2017 and 2019 studies using Fisher’s Exact Tests.

**Results:**

In Case Two and Three, the proportion of correct BP values significantly increased from 2017 (Case Two: 51%; Case Three: 55%) to 2019 (Case Two: 73%; Case Three: 75%). Additionally, proportions of students who reported lower SBP values than setting values were significantly decreased in Case One and Two, with five failing all contexts.

**Conclusions:**

Second-year student BP measurement skills were improved, not only due to repeated Simulation-Based practice but advancing basic science knowledge and mastery experience in ongoing curriculum. Simulation-Based assessment provided an effective tool for evaluating skill retention and proficiency in medical training.

## Introduction

The tools of Simulation-Based Education (SBE) simulate specific physiological characteristics of a patient, providing student practice in developing clinical skills and assessment of student competency in the targeted skills [1,2]. Resources such as simulators and manikins serve as learning tools [2] that can bridge the gap between theory and practice, providing controlled and safe practice opportunities that support development of solid clinical skills and knowledge [3–5]. Use of simulation is a popular strategy designed to enrich the curriculum of traditional teaching methods, and in medical settings, despite the initial large investment for equipment, simulator training can be cost-effective in reducing the need for live subjects[6]. Nevertheless, scientific evidence supporting the effectiveness of SBE may be compromised by methodological limitations, poor research design, limited sample sizes, or inappropriate application of statistical methods[7]. Thus, evidence that demonstrates the advantages of adding SBE to didactic classroom instruction is valuable[8].

Ministry of Education, Culture, Sports, Science and Technology (MEXT) suggests that students develop ‘the ability of Blood Pressure (BP) Measurement by both palpation and auscultation methods at a brachial artery’[9]. Mastery using the auscultatory method of BP measurement is especially challenging for young medical personnel[10]. There is no question that the ability to standardize BP settings in a simulator provides the only truly reliable method of assessing student proficiency and accuracy in BP measurements[1]. During a measurement, we need to deflate the pressure cuff in a certain speed, recognize the Korotkoff sounds on the stethoscope, check the sphygmomanometer pressure values, all of which must be carried out simultaneously[11]. Thus, mastering the skills needs lots of practice and experience[11].

We initiated a study in 2017 to test the effectiveness of SBE in supplementing competency training for first year medical students in BP measurement[12], since it is a basic clinical skill which must be mastered early in medical training[13]. The first experimental segment of the study was initiated the first month of the academic year in Japan[12]. When we began this study in 2017, literature demonstrating the effectiveness of SBE for BP measurement was limited to a study by Leung and Nicholls, which examined whether student performance was influenced by different clinical contexts[14]. Leung’s experimental design assigned each group to a different clinical context[14]. We adapted Leung’s study design so that all groups in the cohort would measure BP on manikin arms for all three clinical contexts[12].

As a second segment of this study, we repeated the experiment in early 2019 with the same cohort, now well into their second year of medical studies. Our research on the effectiveness of SBE was designed as an ongoing prospective longitudinal study following the same cohort of medical students who began their first year at Tokyo Medical University in Japan in 2017.

An overview of the first (2017) segment of the study is as follows: the target was a cohort of 121 students[12]. This segment had two stages. The first stage was the four-day SBE training session from April to May of 2017[12]. On the first and second days, students learned BP measurement using palpation and auscultation, respectively[12]. Procedurally, these skills were taught through lectures, a textbook, and a video that outlined the steps in performing a BP measurement[12]. After the lessons, students practiced taking their peers’ vital signs in laboratory sessions[12]. On the third day, students practiced reading BP on full-size manikin arms, using auscultation for the first time[12]. On the fourth day, students took BP measurements on simulator arms using auscultation, and their skills were then assessed with the Objective Structured Clinical Examination (OSCE)[12]. At this OSCE, instructors gave feedback for all students, additionally retraining students who could not pass the OSCE until they mastered BP reading.

Students were divided into three groups for the second stage of the 2017 experimental segment, which began three months later in September, and from which data were gathered for this study[12]. Students received further classroom training and practiced auscultation on their peers[12]. Each of three groups was assigned to a different experimental day[12]. Students measured BP on three simulator arms assigned three different clinical contexts, described in this paper’s Methods section[12]. The setting BP values of these three manikins were all 120/70 mmHg on all days, regardless of clinical context[12]. Student BP measurement skills were evaluated by Simulation-Based assessment[12]. There were no significant differences in the proportions of correct BP values among the three clinical cases[12]. Further details of the design and results from this study segment were published by this author in 2018, with the proportions of correct BP values as: 55% in Case 1, 51% in Case 2 and 55% in Case 3 although these proportions were slightly different from the numbers in the manuscript[12] since we included no-answers into the denominator in 2017 and this time we did not include no-answers into the denominator.

**Figure 1.**
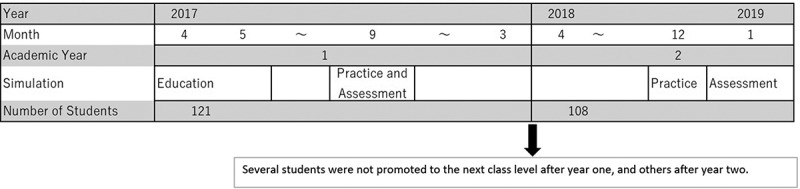
Diagram of flow of simulation-based assessments after simulation-based education and practice

The primary objective of this current study (2019) segment was to assess the improvement of proficiency in BP reading skills of this same cohort, now completing their second year of medical training, after a short Simulation-Based practice. The student assessment method was the same for both years, and proficiency data were compared between 2017 and 2019 for each of the three contexts (Figure 1).

## Methods

The 2019 segment of the study engaged the same cohort of medical students from the 2017 study, numbering 108 due to the exclusion of students who did not advance to the second year. At this point, the students had already studied liberal arts and basic sciences, including anatomy and physiology [12,15].

In the first stage of the second segment, the students underwent BP training with instructors in 2018 that was somewhat enhanced beyond their standard curriculum. Students were divided into five groups and experienced one BP measurement practice during a 3.5-h SBE training, between December 17 and 21, that also included history taking and clinical reasoning. In this session, all students were required to take the BP of either a live subject or a manikin arm under the supervision of instructors. The instructor gave students paper-based learning materials to help them review the procedure of BP measurement, as well as answering student questions and giving feedback for technical problems.

As an ethical consideration, we informed participants of the experimental procedure, that participation was voluntary, and that non-participation would not affect grades. Students signed a consent form when they agreed to participate. In addition, both students and the papers they submitted were anonymous to the research team. The Ethical Review Board of Tokyo Medical University (No. SH3824), Tokyo, Japan, approved this study.

### Data collection in 2019

In the second stage of the 2019 segment of the study, the cohort of 108 students were first divided into four groups (n = 32, 24, 24, and 28). The first two groups, with 32 and 24 students, were tested together on January 15. On January 16, the latter two groups, with 24 and 28 students, were tested in the same manner as the day before. The same procedures for the experiment used in 2017 were followed for both days in 2019, with the exception of the initial BP setting values, which were set differently for each manikin and each day. No participants took part in this experiment more than once in one year. Study design was not revealed to participants, who simply followed instructions.

**Figure 2.**
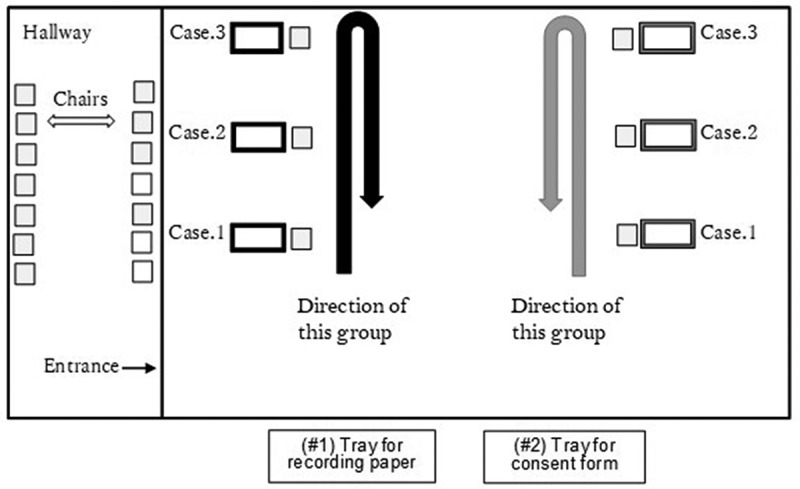
Diagram of layout and procedure for cohort of second-year medical students assessing BP measurement skills in 2019

As the concrete process of data collection: (1) An instructor handed each student a consent form and an anonymous recording paper with three spaces to write down three blood pressure values. (2) Two students, one from each group, entered the room. There were two rows of manikin arms, with both assigned the same series of three context cases. One row was assigned to each group. The two students sat in the front of simulator arm of their Case One scenario of their assigned row. (3) Students read the description of the clinical context assigned that simulator arm. (4) Using auscultation, students measured the BP of the manikin arms and recorded the resulting BP values on the paper anonymously. (5) Students then moved to each of the next two cases and repeated the process. (6) The recording paper was returned to an instructor (Figure 2).

Throughout our Simulation-Based Experiments in 2017 and 2018, three manikin arms (Blood Pressure Trainer, Atsuhime, Kyoto Kagaku Co., Ltd., Kyoto, Japan) were used. The authors followed instrument instructions to calibrate the manikin arm both just before, and one hour after starting, the student readings to ensure accuracy. The diaphragm of a dual-headed stethoscope was used, with BP measurements recorded in mmHg.

### Clinical cases

The three clinical contexts in both rows provided to students measuring the BP of the three manikins were identical to the contexts of the 2017 experiment: Case One was a 20-year-old male college student who was healthy except for pollinosis; Case Two was an 18-year-old female high school student diagnosed with hypotension, presenting with occasional vertigo or a feeling of floating, and one episode of loss of consciousness thrice in morning assembly; and Case Three was an 80-year-old male with a 30-year history of diabetes and hypertension. Typically treated with oral medications, he was admitted this time for glycemic control into the Department of Endocrinology and Metabolism.

Systolic (SBP) over diastolic (DBP) blood pressures were set to the following values:

Day One, First Row: Case One: 140/90, Case Two: 130/80, and Case Three: 120/70;

Day One, Second Row: Case One: 100/60, Case Two: 120/70, and Case Three: 100/60;

Day Two, First Row: Case One: 130/80, Case Two: 100/60, and Case Three: 120/70;

Day Two, Second Row: Case One: 100/60, Case Two: 140/90, and Case Three: 130/80. Within the same day, during recalibrations, each manikin maintained the same BP originally set. SBP and DBP reported by students within ± 5 mmHg of the target settings were considered correct BP values (i.e., an error of more than ± 5 mmHg was not permitted for either value).

### Analysis method in 2019

First, the proportion of correct to incorrect BP reading in 2019 were compared by context case (Table 1). Second, the proportions of correct BP values of the three cases by year (2017 and 2019) were compared (Table 2). Third, SBP and DBP were stratified into three groups: lower than setting value, correct, and higher than setting value, and each of the three groups’ proportions were compared by years (2017 and 2019) and the three clinical contexts (Table 3).

**Table 1.** Proportions of correct answers in a blood pressure measurement practical exam by case of clinical context for a cohort of 108 second-year medical students with first-year SBE training and a second-year review (2019) (Fisher’s Exact Tests)

Variables	Total	Correct	Incorrect	*p*-value
Relationship by Case (Clinical Context)				
Participants n (%)	107 (100)	229 (71)	92 (29)	.37
Case				
One (Normal)	107 (100)	71 (66)	36 (34)	
Two (Hypotension)	107 (100)	78 (73)	29 (27)	
Three (Hypertension)	107 (100)	80 (75)	27 (25)

**Table 2.** The comparisons of proportions of correct answers in a blood pressure measurement assessment for a cohort of medical students by clinical context case between 2017 and 2019 (Fisher’s Exact Tests)

Variables	Total	Correct	Incorrect	*p*-value
Relationship by Year				
Case One (Healthy)				
Participants n (%)	194 (100)	119 (61)	75 (39)	14
Year				
2017	87 (100)	48 (55)	39 (45)	
2019	107 (100)	71 (66)	36 (34)	
Case Two (Hypotension)				
Participants n (%)	202 (100)	126 (62)	76 (38)	<.01
Year				
2017	95 (100)	48 (51)	47 (49)	
2019	107 (100)	78 (73)	29 (27)	
Case Three (Hypertension)				
Participants n (%)	202 (100)	132 (65)	70 (35)	<.01
Year				
2017	95 (100)	52 (55)	43 (45)	
2019	107 (100)	80 (75)	27 (25)

**Table 3.** Comparison by clinical context case and by years (2017 and 2019) of the proportions of Systolic (SBP) and Diastolic (DB) Blood Pressure values stratified by the variation from the setting value into three categories (Fisher’s Exact Tests)

Variables	Total	Lower	Correct	Higher	*p*-value
**Systolic Blood Pressure (SBP)**					
Relationship by Year					
Case One (Healthy)					
Participants n (%)	194 (100)	42 (22)	137 (71)	15 (7.7)	<.05
Year					
2017	87 (100)	26 (30)	55 (63)	6 (7.0)	
2019	107 (100)	16 (15)	82 (77)	9 (8.4)	
Case Two (Hypotension)					
Participants n (%)	202 (100)	58 (29)	137 (68)	7 (3.5)	<.01
Year					
2017	95 (100)	39 (41)	53 (56)	3 (3.2)	
2019	107 (100)	19 (18)	84 (79)	4 (3.7)	
Case Three (Hypertension)					
Participants n (%)	202 (100)	31 (15)	151(75)	20(9.9)	.09
Year					
2017	95 (100)	20 (21)	65 (68)	10 (11)	
2019	107 (100)	11 (10)	86 (80)	10 (9.4)	
**Diastolic Blood Pressure (DBP)**					
There were no significant differences in the three stratified groups					

All analyses were carried out using the statistical software package SAS (version 9.4; SAS Institute, Cary, NC, USA), with *p* <.05 considered statistically significant.

## Results

The participation rate of the target cohort for this experiment was 100%. However, one participant on the first day wrote the SBP and DBP blood pressure values in reverse. Therefore, we excluded this participant from the analysis for a total of 107 responses analyzed. Results of Fisher’s Exact tests showed there were no significant differences in the proportions of correct BP values among three cases in the 2019 experiment (Case One: 66% [71/107]; Case Two: 73% [78/107]; Case Three: 75% [80/107]) (Fisher’s Exact Testχ^2^ (2, N = 321) = 2.04, *p* = .37) (Table 1). Additionally, the proportion of correct BP values in 2019 compared with 2017 by Case significantly increased in Case Two from 51% to 73% (Fisher’s Exact Testχ^2^ [1, N = 202] = 10.7, *p* < .01) and Case Three from 55% to 75% (Fisher’s Exact Testχ^2^ [1, N = 202] = 8.9, *p* < .01) (Table 2). When we categorized SBP and DBP into three groups (Lower, Correct, and Higher) and compared the proportions of these three groups among three cases between years 2017 and 2019, the proportions of those who reported lower SBP values than setting values were significantly decreased from 30% to 15% in Case One (Fisher’s Exact Testχ^2^ [2, N = 194] = 6.3, *p* < .05) and from 41% to 18% in Case Two (Fisher’s Exact Testχ^2^ [2, N = 202] = 13.4, *p* < .01) (Table 3). The proportions of three groups of DBP were not significantly different among the three cases between the two experimental years (*p* > .05). However, five students in 2019 failed in all three cases.

## Discussion

Despite some student failures, overall, BP measurement skills were significantly improved after the SBE training and practice in the medical students’ second year compared to the first year, especially in hypotension and hypertension cases. The proportion of students who reported lower BP values than the setting value significantly decreased in healthy and hypotension cases. Students became more adept at detecting Korotkoff sounds correctly.

Repeated Simulation-Based BP measurement practice can be a valuable tool for skill retention and improvement, and gives learners mental space to perform clinical skills correctly. Since we did not confirm that every student mastered BP reading, the session we provided in 2019 was not a typical training. However, several recent studies in medical education have reported the advantageous aspects of repeated SBE trainings[16–18]. Increased cardiopulmonary resuscitation (CPR) training repetition on a simulator with the concomitant improvement of quality in CPR performance was associated with a positive effect on the rescuer’s belief in cardiopulmonary resuscitation, according to a previous study[17]. Another study reported that repeated training on the Virtual Haptic Back (VHB) contributed to substantial retention of palpatory skills[18]. In addition, Abe et al. demonstrated the effectiveness of repeated simulation training in ureterorenoscopy to reduce anxiety and improve confidence in medical students[16].

Besides the observed effectiveness of repeated SBE practice, the following factors encouraged students to improve their BP measurement skills. First, due to basic science knowledge after learning anatomy and physiology, most students correctly located the radial artery and understood the physiological phenomena affecting BP variation in a single day. As a result, they were not as likely to err on the BP value they measured. Knowledge and understanding of basic biomedical sciences are essential for medical students to conduct clinical practice[19]. Furthermore, basic science knowledge is important for medical student clinical thinking[20]. Second, mastery-experience-related self-efficacy might improve clinical performance of medical students. Palmer indicates that mastery experience is the main source of self-efficacy[21], and high levels of self-efficacy may reduce threat appraisals and anxiety on the examination or the evaluation. Third, stress management may be beneficial to student performance[22]. The second-year medical students had already passed more written and practical exams than when they were tested in 2017 as first-year students. Therefore, they might manage stress and handle tasks more adeptly compared to when they were first-year students. Mastery experience and stress management may play a relevant role in academic success[23]. In their first year, students might measure BP as part of a motor skill. In their second year, their BP reading skills can be expected to be associated with more insightfulness and precision because they have studied physiology and anatomy. Simulation-Based practice aids in the recall of BP measurement procedures, and reinforces through mastery experience.

Even after SBE trainings and Simulation-Based practice, some students had difficulties with BP measurements. This disparity of skill retention demonstrates the need for providing repeated SBE trainings and testing for students not optimally developing necessary clinical skills. Sennhenn-Kirchner et al. conducted an experimental study to assess the impact of repeated testing as opposed to repeated training on suturing skill retention in fourth-year dental students[24]. In their study, fourth-year students were either assigned to two sessions of additional skills training (group A) or two sessions of skills assessment with feedback (group B) from instructors[24]. The OSCE performance was significantly better in group B than group A for suturing[24]. Repeated skills training and/or assessments in which all students can achieve competence can help to guarantee physician quality.

### Limitations

Several limitations were encountered in our study. First, we could not provide SBE training in 2019 similar to that in 2017 due to time restrictions. Second, most students had already been tested in 2017 in our first study in a similar way. Therefore, they might know our purpose, and intentionally not be influenced by clinical contexts. Third, we could not eliminate the influence of holdovers due to the anonymity of students. There is a possibility that those who could not measure BP at all might be holdovers. Those holdovers did not advance to their third year in 2018 and did not participate in the trainings and assessments in 2017. Fourth, several factors such as basic science knowledge, mastery experience, and stress management from learning experiences contributed to student skill mastery, and repeated Simulation-Based practice may have been a less essential factor in skills improvement. More useful data might arise from a qualitative survey investigating student feedback regarding contributors to their skills. Fifth, although the BP measurement practice in 2018 was mandatory, we could not standardize the amount of time students practiced or repeated BP measurements. Sixth, results were not generalized, because participants were all second-year medical students at one medical school. Finally, we did not know whether students had opportunities to take BP measurements outside our practical trainings, which might also influence improvement of skills of BP measurement.

## Conclusions

We found that after SBE and simulation-based practice, second-year medical students could measure BP more correctly and recognized Korotkoff sounds more precisely than when they were in the first year. However, we cannot conclude that the SBE and simulation-based practice were entirely or partially responsible for skill improvements, since basic science knowledge, mastery experience, and stress management skills learned through multiple exams and practical trainings also strengthened skills. In the present experiment, we can say that simulators are useful and effective in assessment of student BP measurement skills. Additionally, even after SBE and practice, some students still have difficulties with BP reading. Therefore, repeating simulation-based assessment to help all students master BP measurement, and receiving feedback from instructors, is essential for optimal clinical skills.
